# circ-SIRT1 Promotes Colorectal Cancer Proliferation and EMT by Recruiting and Binding to eIF4A3

**DOI:** 10.1155/2021/5739769

**Published:** 2021-10-08

**Authors:** Xiangjie Wang, Shuang Liu, Bin Xu, Yabin Liu, Peng Kong, Changlin Li, Binghui Li

**Affiliations:** ^1^Department of General Surgery, Fourth Hospital of Hebei Medical University, Shijiazhuang, Hebei, China; ^2^Department of Biochemistry and Molecular Biology, Basic Medical College, Hebei Medical University, Shijiazhuang, Hebei, China

## Abstract

Circular RNA (circRNA), a recently identified type of endogenous noncoding RNA, has been implicated in the occurrence and development of a variety of tumors; however, whether circ-SIRT1, derived from pre-mRNA of the parental *SIRT1* gene, is involved in colorectal cancer (CRC) remains unknown, as do the potential underlying mechanisms. The expression of circ-SIRT1 in CRC cells and tissue was detected by RT-qPCR. Colony formation and Cell Counting Kit-8 assays were used to evaluate the effect of circ-SIRT1 knockdown on the proliferative ability of CRC cells. Wound healing and Transwell assays were used to assess the effect of circ-SIRT1 knockdown on the migratory and invasive capacity of CRC cells. RNA immunoprecipitation and RNA pull-down assays were employed to validate the binding of circ-SIRT1 to EIF4A3. Western blot was used to identify the changes in the expression of EIF4A3 and EMT-related proteins. The RT-qPCR results showed that circ-SIRT1 was highly expressed in CRC cells and tissue and was positively correlated with the depth of tumor invasion. Knocking down circ-SIRT1 inhibited the proliferation and invasion of CRC cells and EMT. We further found that EIF4A3 could bind to circ-SIRT1, and that overexpressing circ-SIRT1 decreased the abundance of EIF4A3 at the mRNAs of the EMT marker proteins N-cadherin and vimentin. Combined, our findings suggested that circ-SIRT1 regulates the expression of EMT-related proteins by preventing EIF4A3 recruitment to the respective mRNAs. Our results further indicate that circ-SIRT1 functions as an oncogene in CRC by promoting the proliferation, invasion, and EMT of CRC cells through the circ-SIRT1/EIF4A3/N-cadherin/vimentin pathway.

## 1. Introduction

Colorectal cancer (CRC) is the third most commonly diagnosed malignant tumor and the fourth most deadly, being responsible for approximately 600,000 deaths worldwide every year [1, 2]. CRC also ranks fifth as the leading cause of cancer-related deaths in China [3]. Although the 5-year survival rate for CRC patients has improved with the continuous advances in treatment methods, postoperative recurrence and distant metastasis remain the main causes of death among patients with this cancer [4, 5]. It is well known that epithelial cells can undergo an epithelial-to-mesenchymal transition (EMT), which facilitates their infiltration into surrounding tissues and enhances their migratory ability [6]. EMT, which is closely related to tumor proliferation and metastasis [7], is considered to be the main cause of the spread of CRC cells to distant organs of the body [8]. These observations highlight the urgent need to identify the molecular targets associated with the proliferative and metastatic ability of CRC cells and thereby improve the therapeutic options for the treatment of patients with CRC.

Circular RNAs (circRNAs) are endogenous noncoding RNAs ubiquitous across species [9]. Unlike linear RNAs, circRNAs have no 5′-cap structure or 3′ polyA tail, which renders them resistant to hydrolysis by exonucleases and endows them with high stability in the cellular environment. Although circRNAs were first identified in plant viruses as early as 1976, they were initially considered to be a product of erroneous splicing events [10]. However, it is now known that circRNAs play important regulatory roles in the occurrence and development of a variety of cancers, including acting as miRNA sponges and regulating a number of transcriptional and translational processes [11]. Has_circ_0009361 was reported to act as a sponge for micro RNA- (miR-) 582, which positively regulates the expression of its target gene, *APC2*, thereby inhibiting the growth, invasion, and metastasis of CRC cells [12]. Additionally, circ-CSE1L can reportedly inhibit PCNA protein expression and, consequently, CRC cell proliferation [13]. Nevertheless, the functions of many circRNAs in CRC remain uncharacterized.

The sirtuins (SIRTs) comprise a family of mammalian NAD^+^-dependent histone deacetylases. SIRT1, a member of the sirtuin family [14], is a human ortholog of yeast SIR2 (silent information regulator 2) protein [15, 16] known to be involved in numerous cellular processes, including energy metabolism, proliferation and senescence, inflammatory responses, neuroprotection, and tumorigenesis [17]. Human SIRT1 is related to tumor proliferation, metastasis, and invasion in esophageal cancer [18], CRC [19], and breast cancer [20]. A recent study showed that circ-SIRT1 also plays an important role in atherosclerosis and neointima formation [21]. However, a search of circBase revealed that relatively little is known about the CRC-related function of circ-SIRT1, which is derived from the *SIRT1* gene.

In this study, we sought to determine the expression and function of circ-SIRT1 in CRC, as well as identify the underlying mechanisms. The results showed that circ-SIRT1 is highly expressed in CRC tissues, which enhances the proliferation, invasion, and EMT of CRC cells. In addition, we found that circ-SIRT1 can bind to eukaryotic translation initiation factor 4A3 (EIF4A3), thereby blocking the inhibitory effect of EIF4A3 on EMT and promoting the proliferation and invasion of CRC cells. Our results suggest that circ-SIRT1 may represent a novel target for CRC diagnosis and treatment.

## 2. Materials and Methods

### 2.1. Clinical Specimens and Data Collection

The clinical specimens used in this study were obtained from patients who underwent surgery for pathologically confirmed CRC at the Second Surgery Department of the Fourth Hospital of Hebei Medical University from August 2020 to January 2021. All the patients signed an informed consent form, and approval for the study was obtained from the Ethics Committee of the hospital. In this study, clinical specimen inclusion and exclusion criteria are as follows: (1) age between 40 and 80 years old, (2) no preoperative radiotherapy or chemotherapy treatment, (3) no distant metastasis, and (4) no recurrence of colorectal cancer. A total of 52 CRC and matched adjacent tissue specimens were quickly placed in liquid nitrogen and stored at −80°C. Detailed clinical data of the patients were recorded, and HE staining on the postoperative tumor tissues by the pathology department.

### 2.2. Cell Culture

FHC, HCT116, and HT29 cell lines were purchased from American Type Culture Collection (ATCC; Manassas, VA, USA). FHC cells were cultured in DMEM (Invitrogen; Carlsbad, CA, USA), while HCT116 and HT29 cells were cultured in McCoy's 5A medium (Gibco; Thermofisher Scientific, Waltham, MA, USA). All the media were supplemented with 10% fetal bovine serum (FBS; Gibco, USA), 100 U/mL penicillin, and 100 *μ*g/mL streptomycin. All cells were cultured in an incubator at 37°C with 5% CO_2_. The medium was changed regularly.

### 2.3. RNA Extraction and RT-qPCR

Total RNA was extracted from clinical tissues (diameter~5 mm) and cells (area~20 cm^2^) using Trizol reagent (Sangon, Shanghai, China). RNA purity was evaluated by spectrophotometry and was used at an OD (260/280 nm) of between 1.9 and 2.1 for the experiments. cDNA was reverse transcribed from the extracted RNA using the M-MLV First Strand cDNA Synthesis Kit (Beyotime, Beijing, China). qPCR was performed using SYBR Green qPCR SuperMix-UDG (Beyotime) in a 7300 Real-Time PCR System (Thermofisher Scientific, Waltham, MA, USA). Amplification was performed in a total volume of 20 *μ*L under the following cycling conditions: an initial denaturation at 95°C for 2 min, followed by 40 cycles at 95°C for 15 s, 60°C for 30 s for annealing, and 72°C for 30 s for extension, followed by dissociation curve analysis. *GAPDH* was used as the reference gene. The following primers (Sangon) were used: circ-SIRT1 5′-AGAGATTGTGTTTTTTGGTGAA-3′ (forward) and 5′-GAAGGTTATTTGGAATTAGTGC-3′ (reverse); GAPDH 5′-ATCTTCCAGGAGCGAGATCCC-3′ (forward) and 5′-TGAGTCTTCCACGATACCAA-3′ (reverse). Relative circ-SIRT1 expression levels of tissues and cell lines were normalized to those of GAPDH and using *Δ*CT and 2-*ΔΔ*CT methods, respectively.

### 2.4. Cell Transfection

The siRNAs targeting circ-SIRT1 and EIF4A3 were synthesized by GenePharma (Shanghai, China). The sequences were si-circ-SIRT1#1: 5′-GCACUAAUUCCAAAUAACCTT-3′ (sense) and 5′-GGUUAUUUGGAAUUAGUGCTT-3′ (antisense); si-circ-SIRT1#2: 5′-CUAAUUCCAAAUAACCUUCTT-3′ (sense) and 5′-GAAGGUUAUUUGGAAUUAGTT-3′ (antisense); si-EIF4A3: AGACAUGACUAAAGUGGAA. The vector for circ-SIRT1 overexpression (Sangon Biotech) was generated by inserting the complete human circ-SIRT1 sequence in the pcDNA3.1 vector. HCT116 and HT29 cells were transfected at approximately 70% confluence using Lipo8000 transfection reagent (Beyotime).

### 2.5. Colony Formation Assay

HCT116 and HT29 cells (500 each) transfected in the logarithmic phase were added to each well of a 6-well plate. After adding 2 mL of McCoy's 5A medium containing 10% FBS, the cells were cultured for 14 days. When colonies were visible to the naked eye, the cells were fixed in 4% paraformaldehyde, stained with crystal violet, photographed (Olympus Corp, Tokyo, Japan), and counted (ImageJ 1.52a, National Institutes of Health, USA).

### 2.6. Cell Counting Kit-8 (CCK-8) Assay

Briefly, approximately 3,000 cells were added to each well of a 96-well plate and cultured for 24, 48, 72, or 96 h. At each time point, 10 *μ*L of CCK-8 reagent (APExBIO, Houston, TX, USA) was added to the cells, followed by incubation at 37°C for 2 h. Absorbance at 450 nm was measured using a microplate reader.

### 2.7. Wound Healing Assay

Three horizontal lines, 5 mm apart, were drawn at the bottom of a 6-well plate. HCT116 and HT29 cells were transfected at approximately 70% confluence. A 200 *μ*L pipette tip was used to make three scratches (wounds) across the cell layer perpendicular to the horizontal lines in the 6-well plate. After washing with PBS, the cells were photographed (0 h) (Olympus Corp.), cultured for 36 h, and then photographed again to determine cell migration and calculate the migration area (ImageJ 1.52a, National Institutes of Health, USA).

### 2.8. Transwell Invasion Assay

Cell invasion ability was measured using Transwell plates. Matrigel (40 *μ*L) (Invitrogen) was applied to the upper chamber and placed in an incubator at 37°C for 24 h. Six hours after transfection, HCT116 and HT29 cells were starved in serum-free medium for 24 h. A total of 1 × 10^5^ cells and 200 *μ*L of serum-free medium were added to the upper chamber of each well that had been precoated with Matrigel, while 600 *μ*L of McCoy's 5A medium containing 10% FBS was added to the lower chamber. After 24 h of culture, the nonmigrated cells in the upper chamber were gently wiped off with a cotton swab. Migrated cells were fixed in 4% paraformaldehyde for 20 min, stained with crystal violet for 20 min, and photographed and counted under an inverted microscope (Olympus Corp).

### 2.9. Western Blot

Total protein was extracted from transfected HCT116 and HT29 cells using lysis buffer. The protein concentration was measured using the Lowry assay. Equal amounts of protein (40 *μ*g) were separated by 10% sodium dodecyl sulfate–polyacrylamide gel electrophoresis and transferred onto polyvinylidene fluoride membranes. After blocking in 5% skimmed milk for 2 h, the membranes were incubated with antibodies (all from Abcam, USA) targeting N-cadherin (280375, mouse monoclonal), E-cadherin (232410, rabbit monoclonal), vimentin (92547, rabbit monoclonal), EIF4A3 (32485, rabbit polyclonal), and GAPDH (9485, rabbit polyclonal) overnight at 4°C. After washing off the primary antibody, the membranes were incubated with the corresponding secondary antibodies at 37°C for 1 h. The films were scanned using a chemiluminescence imaging system.

### 2.10. RNA Immunoprecipitation Assay (RIP)

The RIP assay was performed using a RIP kit from Millipore (Massachusetts, USA). Cells were lysed and incubated with magnetic beads and anti-EIF4A3 antibody, with nonspecific IgG serving as a control. RNA was extracted from the magnetic beads and analyzed by RT-qPCR.

### 2.11. RNA Pull-Down Assay

Lysis buffer was added to HCT116 and HT29 cells, and total protein was extracted by freezing and thawing three times in liquid nitrogen. The circ-SIRT1 probe (Sangon) was added to generate a circ-SIRT1/protein complex which was incubated with streptavidin-complexed magnetic beads at 4°C for 3 h. The magnetic beads were extracted, and EIF4A3 protein expression was detected by western blot.

### 2.12. Statistical Analysis

All data were analyzed using SPSS 25.0 (IBM, USA) and GraphPad Prism 8 (GraphPad Software, La Jolla, CA, USA). The results are expressed as means ± standard error of the mean (SEM). The *t*-test was used for comparisons between two groups, and the independent samples *t*-test was used to assess the clinical significance of circ-SIRT1 in CRC. A *P* value of < 0.05 was defined as statistically significant.

## 3. Results

### 3.1. circ-SIRT1 Expression Was Upregulated in CRC and Was Positively Correlated with the Depth of Tumor Invasion

**Figure 1:**
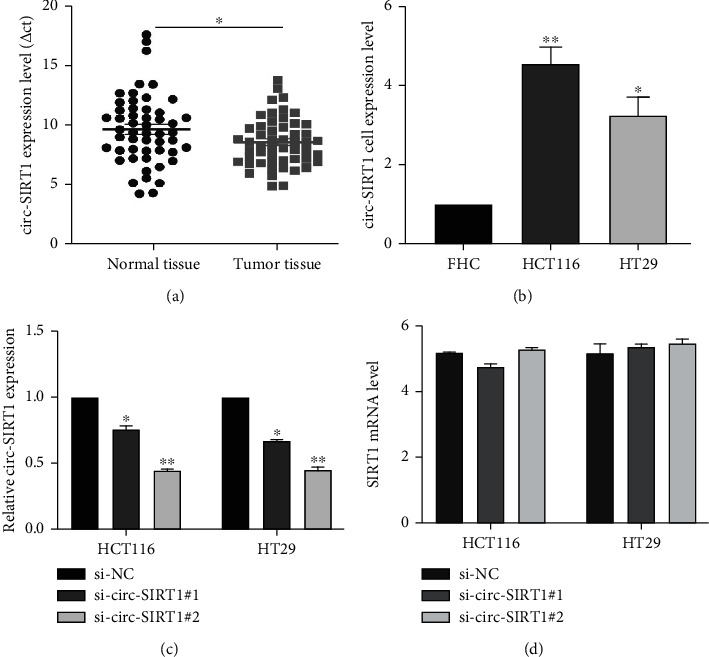
circ-SIRT1 was upregulated in colorectal cancer tissues and cells. The relative expression of circ-SIRT1 in human CRC tissues and matched normal colorectal tissues (a). The expression level of circ-SIRT1 in FHC, HCT116, and HT29 cell lines (b). circ-SIRT1 was effectively knocked down in HCT116 and HT29 cells transfected with si-circ-SIRT1 (c). Expression of SIRT1 in HCT116 and HT29 cells transfected with si-circ-SIRT1 (d). ^∗^ stands for *P* < 0.05, ^∗∗^ stands for *P* < 0.01.

In this study, we collected 52 human CRC tissues and matched normal colorectal tissues. Compared with that in normal tissues, the expression of circ-SIRT1 was significantly upregulated in CRC tissues (*P* < 0.05) (Figure 1(a)). To verify this result, we used RT-qPCR to measure the expression level of circ-SIRT1 in FHC, HCT116, and HT29 cells. The results showed that circ-SIRT1 was highly expressed in HCT116 and HT29 cells when compared with FHC cells (*P* < 0.05) (Figure 1(b)), which was consistent with the results obtained with the tissue samples. Analysis of clinical data indicated that the expression level of circ-SIRT1 was positively correlated with the depth of tumor invasion (*P* < 0.05), but had no correlation with gender, age, tumor size, tumor location, or any other patient characteristic (*P* > 0.05) (Table 1).

### 3.2. circ-SIRT1 Was Effectively Knocked down in HCT116 and HT29 Cells Transfected with si-circ-SIRT1

To manipulate the circ-SIRT1 levels in CRC cells, we transfected HCT116 and HT29 cells with si-circ-SIRT1. The results showed that circ-SIRT1 was effectively knocked down and that the knockdown efficiency of siRNA-circ-SIRT1#2 was greater than that of siRNA-circ-SIRT1#1 (*P* < 0.01) (Figure 1(c)). Next, we examined whether knocking down circ-SIRT1 affected the expression level of SIRT1. No significant change in the level of *SIRT1* mRNA was detected (Figure 1(d)).

### 3.3. circ-SIRT1 Knockdown Inhibited the Proliferation of CRC Cells

**Figure 2:**
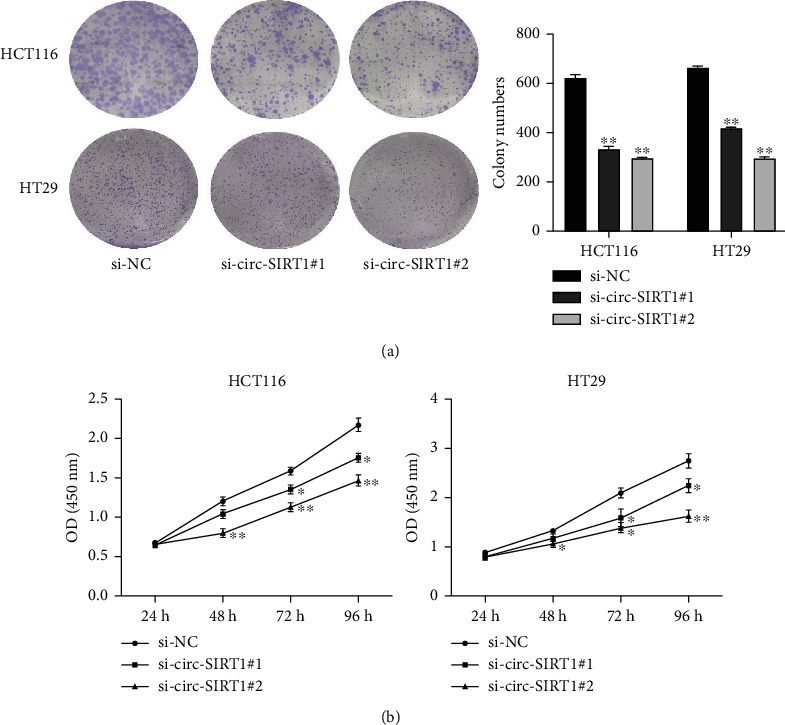
The effect of downregulation of circ-SIRT1 expression on the proliferation of HCT116 and HT29 cells. Downregulation of cirC-SIRT1 expression inhibited the proliferation of HCT116 and HT29 cells (a) and (b). ^∗^ stands for *P* < 0.05, ^∗∗^ stands for *P* < 0.01, ^∗∗∗^ stands for *P* < 0.001.

Because we found that circ-SIRT1 expression was upregulated in CRC, we next explored the biological significance of circ-SIRT1 in tumorigenesis. For this, we first knocked down circ-SIRT1 and determined the effect on cell proliferation using colony formation and CCK-8 assays. The results of the colony formation assay showed that, compared with controls, the proliferative ability of HCT116 and HT29 cells was significantly inhibited when the circ-SIRT1 expression was depleted (Figure 2(a)). Similar results were obtained with the CCK-8 assay (Figure 2(b)).

### 3.4. circ-SIRT1 Knockdown Inhibited the Migration and Invasion of CRC Cells

**Figure 3:**
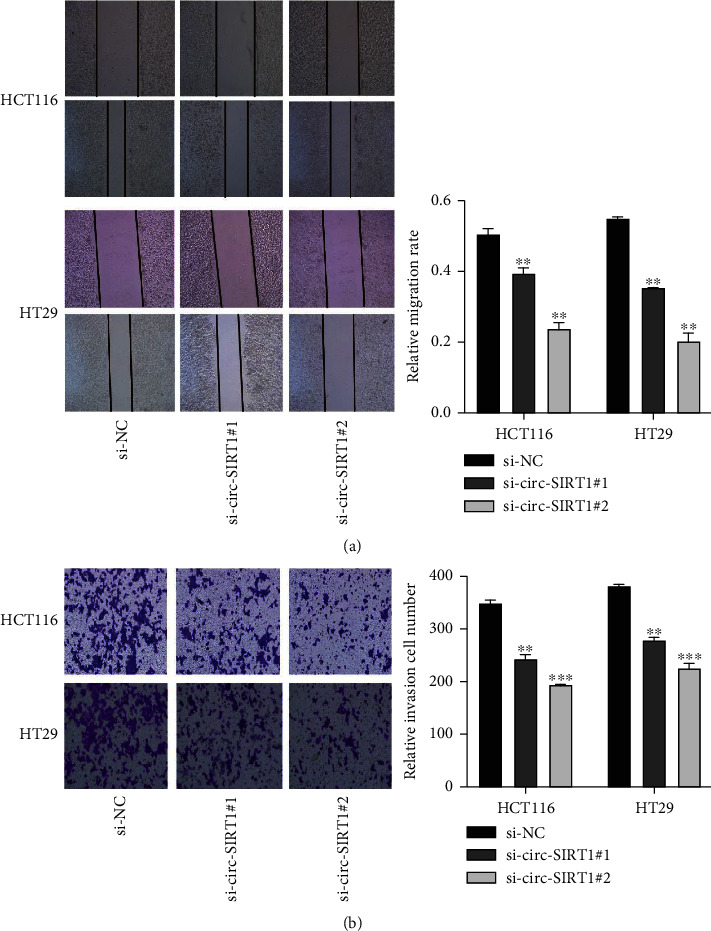
The effect of si-RNA knocking down circ-SIRT1 on the migration and invasion of HCT116 and HT29 cells. Knockdown of circ-SIRT1 has an inhibitory effect on the migration ability of HCT116 and HT29 cells (a). Knockdown of circ-SIRT1 inhibits the invasion ability of HCT116 and HT29 cells (b). ^∗^ stands for *P* < 0.05, ^∗∗^ stands for *P* < 0.01.

**Figure 4:**
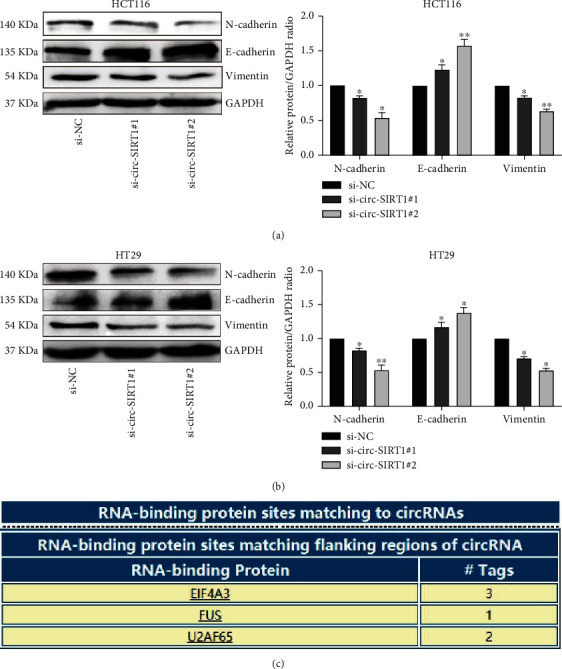
The relationship between low expression of circ-SIRT1 and EMT. Knockdown of circ-SIRT1 can inhibit the EMT process by inhibiting the expression of mesenchymal marker protein (a) and (b). Predict the proteins that can bind to circ-SIRT1 through circInteractome (c).

The migratory and invasive abilities of CRC cells were evaluated through wound healing and transwell assays. We found that knocking down circ-SIRT1 could effectively inhibit the migration and invasion of HCT116 and HT29 cells (Figures 3(a) and 3(b)) when compared with upregulated circ-SIRT1 cells. Because EMT endows stationary epithelial cells with the ability to migrate and invade adjacent tissues, we assessed whether the expression of EMT marker proteins was altered following circ-SIRT1 knockdown. The results showed, in circ-SIRT1-depleted cells, N-cadherin and vimentin expression was downregulated, whereas that and of E-cadherin was upregulated (Figures 4(a) and 4(b)).

### 3.5. circ-SIRT1 Could Bind to EIF4A3

**Figure 5:**
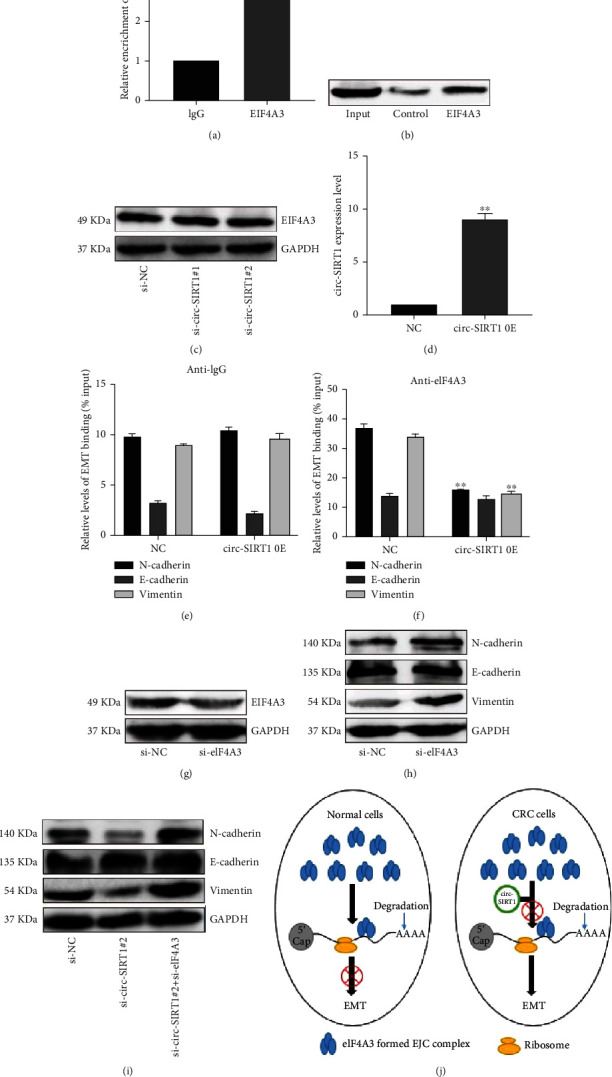
circ-SIRT1 promotes EMT by binding to eIF4A3. RIP and RNA pull-down assays demonstrated the binding of circ-SIRT1 to eIF4A3 (a) and (b). The expression of EIF4A3 was not regulated by cirC-SIRT1 (c). The expression of circ-SIRT1 after transfecting HCT116 cells with overexpression vector (d). Anti-IgG antibody was used as a negative control. After overexpression of circ-SIRT1, enrichment of EMT-related proteins mRNA in immunoprecipitation of anti-EIF4A3 RNA (e) and (f). Downregulation of EIF4A3 expression promotes the expression of EMT-related proteins N-cadherin and vimentin (g) and (h). Knockdown of EIF4A3 can reverse the inhibitory effect of circ-SIRT1 on EMT (i). Schematic diagram of circ-SIRT1 combined with eIF4A3 to regulate the proliferation of colorectal cancer cells (j). ^∗^ stands for *P* < 0.05, ^∗∗^ stands for *P* < 0.01.

Accumulating evidence has indicated that noncoding RNAs can bind to specific proteins and influence tumor initiation/progression [11, 13]. We used the CircInteractome web tool (https://circinteractome.nia.nih.gov) to predict the proteins that could bind to circ-SIRT1 and selected the highest ranked, namely, EIF4A3, for follow-up analysis (Figure 4(c)). The RIP assay showed that circ-SIRT1 could interact with EIF4A3 (Figure 5(a)) and further confirmed circ-SIRT1/EIF4A3 binding using RNA pull-down experiments (Figure 5(b)). To investigate whether the expression of EIF4A3 is regulated by circ-SIRT1, we detected the expression of EIF4A3 after knocking down circ-SIRT1; no difference was found in EIF4A3 expression (Figure 5(c)). Combined, these data suggested that the EIF4A3/circ-SIRT1 combination may play a regulatory role in CRC proliferation.

### 3.6. circ-SIRT1 Regulated the Expression of EMT-Related Proteins by Preventing the Recruitment of EIF4A3 to Their Respective mRNAs

EIF4A3 is involved in mRNA quality control before translation initiation. Studies have shown that EIF4A3 can modulate neuronal protein expression and synaptic strength [22]. Here, we hypothesized that the circ-SIRT1/EIF4A3 combination might affect EIF4A3 abundance at the mRNA of EMT marker proteins. After overexpressing circ-SIRT1 (Figure 5(d)), we employed an RIP assay to detect EIF4A3 enrichment at the mRNA of EMT-related proteins, with anti-IgG antibody serving as the negative control. The results showed that less N-cadherin and vimentin mRNA precipitated with anti-EIF4A3 compared with that in the control situation (Figures 5(e) and 5(f)). These findings suggested that circ-SIRT1 may regulate the expression of EMT-related marker proteins by preventing the recruitment of EIF4A3 to the respective mRNAs. To test this, we first transfected HCT116 cells with si-EIF4A3. As shown in Figure 5(g), knocking down EIF4A3 upregulated the expression of the EMT marker proteins N-cadherin and vimentin but did not affect that of E-cadherin (Figure 5(h)). Then, we cotransfected si-EIF4A3 and si-RNA-circ-SIRT1#2 into HCT116 cells (Figure 5(i)) and found that downregulating EIF4A3 could reverse the inhibitory effect of downregulating circ-SIRT1 on EMT. These results were consistent with our previous studies.

## 4. Discussion

circRNAs are no longer regarded as “noise” resulting from mRNA missplicing events. Accordingly, increasing attention has focused on their biological significance and function, especially in malignant tumors. For instance, circ-ITCH expression is downregulated in CRC, where it acts as a tumor suppressor by inhibiting CRC cell proliferation and metastasis [23]. In contrast, circ-HIPK3 and circ-PPP1R12A are significantly upregulated in CRC and function as oncogenes through the promotion of tumor cell proliferation and metastasis [24, 25]. Recurrence and metastasis are the main causes of death in patients with CRC, and elucidating the molecular mechanism underlying CRC proliferation and metastasis in the earliest possible timeframe could provide more therapeutic options for the treatment of this disease.

SIRT1 has been implicated in the regulation of cell metabolism and senescence [26]. Additionally, SIRT1 expression has been reported to be upregulated in breast [20], lung [27], and prostate cancer [28], as well as CRC [19, 29]. Here, we found that the expression level of circ-SIRT1 was upregulated in CRC tissues compared with that in paired, adjacent nontumor tissue samples. Analysis of the postoperative pathology of clinical patients indicated that, compared with patients with low circ-SIRT1 expression, those with high expression of circ-SIRT1 had greater depth of tumor invasion. HE staining on the postoperative tumor tissues by the pathology department of our hospital confirmed this observation. However, we did not find a significant correlation between circ-SIRT1 and TNM staging, lymph node metastasis, or other tumor characteristics, which may have been due to the small sample size in our study. Further studies are needed to confirm these results. Following circ-SIRT1 knockdown, we observed that the proliferative, migratory, and invasion abilities of HCT116 and HT29 cells were suppressed compared with those of control cells. These results suggest that circ-SIRT1 acts as an oncogene in CRC by promoting the proliferation, migration, and invasion of CRC cells.

EMT is an important component in tumor proliferation and metastasis, accelerating the migration and invasion of tumor cells [8, 30]. For instance, lncRNA HIF1A-AS2 positively affects the progression of CRC and EMT formation through the regulation of miR-129-5p and DNMT3A [31]. Meanwhile, circPTPN22 inhibits the proliferation and metastasis of gastric cancer cells by inhibiting the EMT pathway through competitive miRNA binding [32]. To evaluate whether circ-SIRT1 knockdown affects EMT, thereby inhibiting cell migration and invasion, we measured the expression of EMT marker proteins in CRC cells depleted of circ-SIRT1. The results showed that the expression of the EMT marker proteins N-cadherin and vimentin was decreased, while that of E-cadherin was increased, indicating that the loss of circ-SIRT1 expression can result in EMT inhibition.

Several studies have reported on the interaction between circRNAs and proteins in tumors [32, 33]. Here, we used the CircInteractome web tool to predict the proteins that could bind to circ-SIRT1 and selected the highest-ranked one, EIF4A3, for further investigation. We confirmed the binding of circ-SIRT1 to EIF4A3 using RIP and RNA pull-down experiments. Additionally, we found that circ-SIRT1 did not regulate EIF4A3 expression, which implied that EIF4A3 could instead be recruited by circ-SIRT1 to regulate downstream targets. EIF4A3 is an important component of the exon junction complex (EJC) that is loaded onto mRNAs by pre-mRNA splicing, which triggers nonsense-mediated mRNA decay (NMD) and thereby influences protein translation and expression levels [34, 35]. The binding of lncRNA H19 to EIF4A3 can upregulate the mRNA expression of cyclin E1, cyclin D1, and CDK4, thus, enhancing the proliferative ability of CRC cells [36]. Accordingly, we speculated that the circ-SIRT1/EIF4A3 combination may affect EIF4A3 abundance at the mRNAs of EMT-related proteins. Our idea was verified using a RIP assay, with the results showing that N-cadherin and vimentin mRNA precipitation levels were reduced in the anti-EIF4A3 antibody/RNA immunoprecipitation fraction, whereas no significant change was detected for E-cadherin mRNA, and this may be caused by different results in different mRNA regions of EJC, possibility that warrants further investigation. There is evidence to show that *EIF4A3* gene knockout results in a significant increase in activity-regulated cytoskeletal-associated protein (ARC) mRNA and protein expression levels in somatic cells and dendrites [22]. To further validate the underlying mechanisms, we transfected HCT116 cells with si-EIF4A3 or with si-EIF4A3 plus si-RNA-circ-SIRT1#2 and found that both could enhance N-cadherin and vimentin expression, while si-EIF4A3 could reverse the inhibitory effect of si-circ-SIRT1 on EMT (Figure 5(j)).

## 5. Conclusions

In conclusion, our data suggested that circ-siRT1 hindered the recruitment of EIF4A3 to mRNAs of EMT-related proteins, thereby promoting EMT progression and the proliferation of CRC cells. This is the first study to demonstrate that the circ-SIRT1/EIF4A3/N-cadherin/vimentin axis promotes the proliferation and EMT of CRC cells, providing a novel therapeutic target and strategy for the treatment of this cancer. However, more work is needed to understand the mechanism underlying the regulatory effect of circ-SIRT1/EIF4A3 on E-cadherin expression.

## Figures and Tables

**Table 1:** The relationship between the expression level of circ-SIRT1 in colorectal cancer and clinical factors.

Clinical factors	Group	No. of patients	Mean ± S.E.	*t* value	*P* value
Gender	Male	28	1.29 ± 0.087	1.28	0.21
	Female	24	1.46 ± 0.104		
Age	≤60	22	1.32 ± 0.102	0.60	0.56
	>60	30	1.40 ± 0.091		
Tumor size	≤5 cm	24	1.42 ± 0.103	0.70	0.49
	>5 cm	28	1.32 ± 0.090		
Invasive depth	*T*1 + *T*2	21	1.57 ± 0.111	2.66	0.01^∗^
	*T*3 + *T*4	31	1.23 ± 0.076		
TNM stage	I–II	28	1.46 ± 0.096	1.61	0.11
	III–IV	24	1.25 ± 0.090		
Tumor location	Colon	21	1.29 ± 0.101	0.97	0.34
	Rectum	31	1.42 ± 0.090		
Lymph node metastasis	Negative	29	1.45 ± 0.094	1.40	0.17
	Positive	23	1.26 ± 0.094		

^∗^ stands for *P* < 0.05.

## Data Availability

The data used to support the findings of this study are available from the corresponding author upon request.
